# Chikungunya viral load in cerebrospinal fluid and blood is not associated with severity of neurological manifestations in children

**DOI:** 10.1371/journal.pntd.0014112

**Published:** 2026-03-17

**Authors:** Fatiha Najioullah, Rishika Banydeen, Nicolas Garofalo-Gomez, Cristina Santamaria-Dominguez, Martin Savary, Marion Philbert, Raymond Cesaire, Moustapha Dramé, Yves Hatchuel

**Affiliations:** 1 Department of Clinical Research and Innovation, University Hospital of Martinique, Fort de France, France; 2 Department of Virology, University Hospital of Martinique, Fort de France, France; 3 Service of Pediatrics, MFME University Hospital, Fort de France, Martinique, France; 4 EpiCliV Research Unit, University of the French West Indies (Université des Antilles), Fort-de-France, Martinique, France; Faculty of Science, Ain Shams University (ASU), EGYPT

## Abstract

**Background:**

Chikungunya virus (CHIKV) can induce severe neurological manifestations in children. Investigating the role of the viral load (VL) in the blood and cerebrospinal fluid (CSF) could be of interest in understanding the mechanisms that mediate severity. This study aimed to analyze the characteristics of neurological manifestations of CHIKV in young patients at diagnosis and follow-up, with a particular focus on the potential relation between the severity of neurological involvement and the VL in the CSF and blood.

**Methodology/Principal findings:**

We conducted an observational longitudinal retrospective single-center study during the Chikungunya outbreak of 2014 on the French Caribbean Island of Martinique. We included children (excluding newborns) requiring lumbar puncture and who had positive CHIKV RT-PCR in the blood. Blood and CSF VL were assessed, and sociodemographic, clinical and biological characteristics were recorded.

Among 651 children with a positive CHIKV RT-PCR in the blood; 86 were included, of whom 84 had positive RT-PCR in the CSF. Seven children developed probable encephalitis. Neurological manifestations were deemed severe in eight patients (9.3%), intermediate in 11 (12.8%) and non-severe in 67 (77.9%). Mean VL was 9.8 log in blood and 4.9 log in CSF. While mean blood and CSF VL were significantly higher in children aged <1 year, there was no significant association between blood and CSF VL and neurological severity. An initial follow-up carried out on 20 children one to six months after infection, showed good recovery. Additionally, 45 children underwent a neurology consultation 4 years later, of whom 8 (17.8%) presented with neurodevelopmental impairment.

**Conclusion:**

Our results suggest that the CHIKV can invade the CNS at a high level during the acute phase of infection, but does not seem to be associated with the severity of neurological manifestations in children at the acute phase or with long-term cognitive development.

## Author summary

Neurological manifestations including encephalitis may complicate chikungunya infection among children. Hypothesizing that the viral load (VL) in blood and cerebrospinal fluid (CSF) could be of interest in understanding the mechanisms that mediate severity, we conducted a study on children infected by chikungunya virus and who required lumbar puncture during the 2014 Chikungunya outbreak on the Caribbean Island of Martinique. Among 651 children who had a positive RT-PCR in blood, we included 86 who required lumbar puncture, after exclusion of newborns and patients with bacterial meningitis. Neurological manifestations ranged from severe in eight patients (9.3%), intermediate in 11 (12.8%), to non severe in 67 (77.9%). Mean VL was 9.8 log in the blood and 4.9 log in the CSF. An initial follow-up carried out on 20 children one to six months after infection, showed good recovery. Additionally, 45 children underwent a neurology consultation 4 years later, of whom 8 presented with neurodevelopmental impairment. Our results suggest that chikungunya virus can invade the CNS at a high level during the acute phase of infection, without significant association between the viral load in the CSF or blood and the neurological severity of disease and the long-term cognitive development of infected children.

## Introduction

Chikungunya fever (CHIKF) is a vector-borne viral infection that has spread following an endemic-epidemic pattern in several parts of the world since the sixties. In 2005–2006, a large-scale outbreak affected the French island of La Reunion (Indian Ocean) [1]*.* In December 2013, the first indigenous CHIKF cases in the Americas were reported [2,3]. In Martinique, the epidemic started in December 2013 and ended in January 2015. Approximately 36% of the population was affected [4].

The classical clinical manifestations of CHIKF infection are fever, maculopapular rash, and rheumatological manifestations. Different atypical clinical manifestations have also been documented (*e.g.,* cardiovascular or neurological) [5–8] resulting, for some patients, in severe manifestations [5,9]*.* The proportion of patients with neurological involvement varies across studies, and ranges from 0.1% [10] to 16.3% [11]. The most common manifestation is encephalopathy, but encephalitis, Guillain-Barré Syndrome and myeloradiculitis have also been reported [10,12]*.* In adults, encephalitis accounts for up to 55.1% of neurological disorders [11].

In children, the main clinical characteristics of CHIKF are a high prevalence of dermatological and digestive manifestations [13–15], as well as neurological complications ranging from encephalitis and seizures to meningeal syndrome [8,16–18]. According to various studies worldwide, neurological manifestations occur in 14% to 35% of infected children [6,13,19,20]. Moreover, a study from Kenya reported that the incidence of CHIV-associated neurological diseases was higher than cerebral malaria or bacterial meningitis [21]. Indeed, chikungunya virus (CHIKV) is listed by the World Health Organization (WHO) among the pathogens that can induce neurologic manifestations [12]*.*

The results of follow-up studies on children exposed to CHIKV during pregnancy showed that, apart from infection during the intrapartum period, no significant neurodevelopmental delay was observed at the age of two years [22–24]. However, the CHIMERE study, which evaluated the neurodevelopment of children exposed to perinatal CHIKV infection from mother to child, reported a 50% rate of developmental delay in symptomatic infants and a poor neurocognitive outcome in infected children [22].

The work-up of patients with clinical neurological manifestations usually involves the detection of CHIKV using RT-PCR and/or IgM in the cerebrospinal fluid (CSF) and/or in the blood [8,16,18]. However, the utility of measuring the viral load (VL) is controversial. The reported association between VL and clinical features differs across studies [25–29]. To date, the possible relationship of CSF VL with the severity of neurological damage remains unknown. Furthermore, the pathophysiological mechanisms by which CHIKV affects the nervous system have not been fully elucidated. The only three autopsy reports we found in the literature are in favor of the hypothesis of a vascular mechanism secondary to the primary cytokine response [30,31]. However, animal models have shown that after peripheral inoculation, the virus could disseminate into the CNS, via the choroid plexus after viremia, but it does not seem to invade the cerebral parenchyma [32].

In 2024, a vaccine was approved and licensed in several regions of the world [33]. It is recommended for the prevention of disease caused by CHIKV in individuals aged 18 and over. In Europe, the vaccine indication has been extended, and the vaccine is authorized in those aged 12 and over.

The aim of the present study was to analyze the characteristics of neurological manifestations of CHIKV in young patients (under 18 years of age, excluding newborns) at diagnosis and follow-up, with a particular focus on the potential relation between the severity of neurological involvement and the viral load in the CSF and blood.

## Patients and methods

### Ethics statement

All patients were managed in accordance with the amended Declaration of Helsinki (https://www.wma.net/what-we-do/medical-ethics/declaration-of-helsinki) and Good Clinical Practice guidelines. Each child’s legal representative was informed about the study orally and by personal correspondence, giving them the opportunity to explicitly refuse the use of their child’s medical data for research purposes. Oral formal consent was provided by parents or legal guardians of study participants on enrollment. The study was approved by the French National data privacy commission (CNIL, Commission Nationale Informatique et Liberté) under the number N°1897956.

### Study setting

Martinique is a French overseas territory belonging to the Lesser Antilles in the Eastern Caribbean Sea, with a population of approximately 384,000 inhabitants. On 18^th^ December 2013, health authorities reported the first confirmed indigenous CHIKV cases in Martinique, and the outbreak ended in January 2015.

### Study design and patients

We conducted a single-center, observational, longitudinal, retrospective study, investigating the neurological manifestations of CHIKF in children admitted to the University Hospital of Martinique, between 1^st^January 2014 and 31^st^ January 2015. It is the sole hospital for the whole island providing inpatient hospitalization services for sick children.

At the start of the epidemic in Martinique (December 2013), the pediatric team, alerted by the description of neurological involvement during the epidemic in the island of Réunion, issued recommendations for the systematic investigation by lumbar puncture (LP) of all suspected cases of neurological involvement.

For the present study, we included all patients aged more than 10 days and less than 18 years, who consulted the pediatric emergency department or were hospitalized at the University Hospital of Martinique, and who presented with CHIKV infection requiring a lumbar puncture (LP).

In line with guidelines for pediatric management, the indications for lumbar puncture were as follows:

- Before 3 months, mainly microbial testing for suspected sepsis, in line with French guidelines. At this age, neurological manifestations usually cannot be distinguished from sepsis.
- Between 3 and 12 months: suspected sepsis or neurological impairment.
- Between 1 and 5 years of age: neurological impairment, except for isolated seizure
  - For children over 5 years’: neurological impairment including isolated seizure

**Fig 1:**
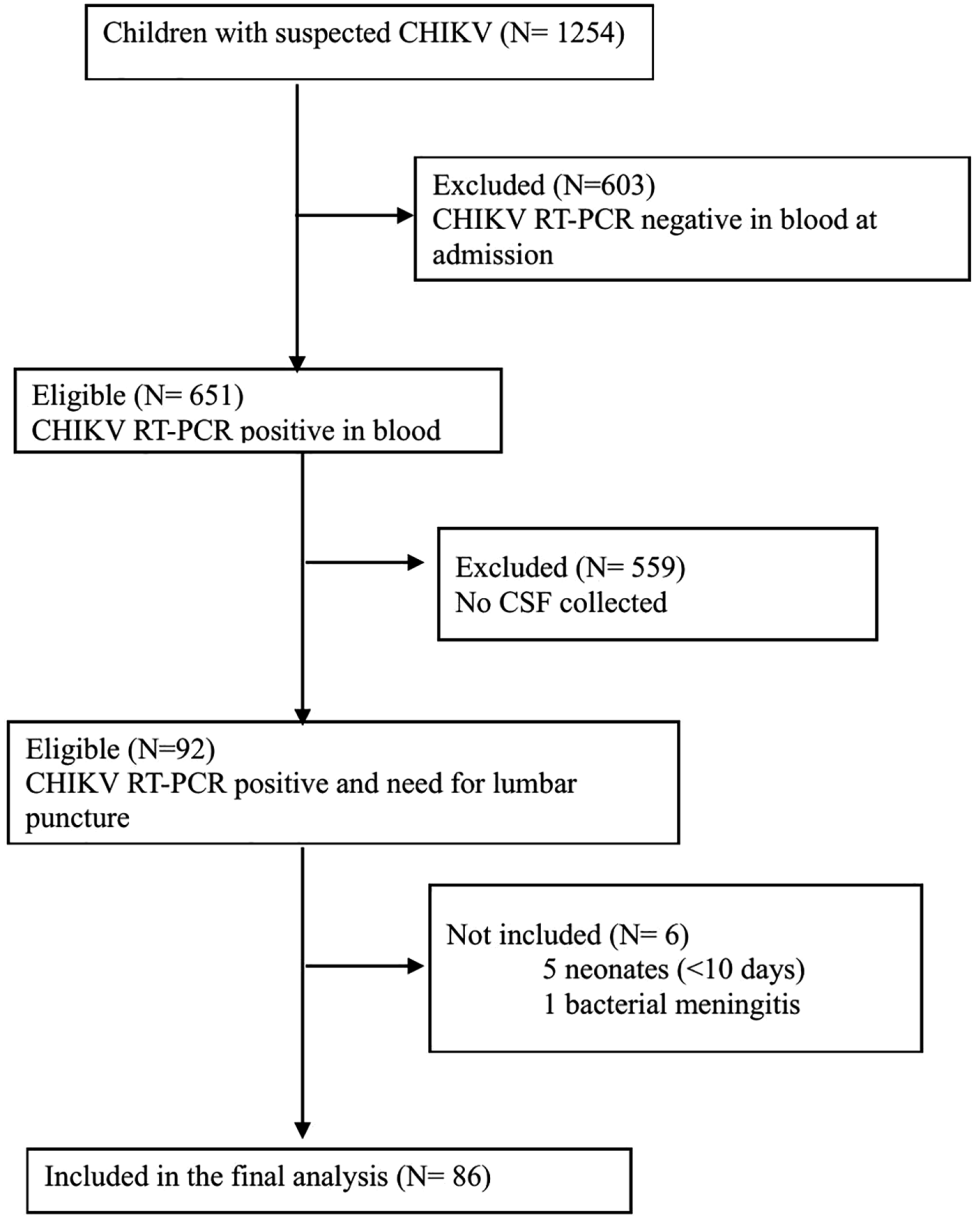
Flow diagram of the study population.

Newborns less than 10 days old were excluded from the analysis to avoid any confusion with maternal-fetal transmission (Fig 1).

Children with bacterial or fungal meningitis were also excluded.

### Patient follow-up

Children aged >10 days and < 3 months, as well as those who presented with severe manifestations during hospitalization for CHIKV infection, were followed up between 1 and 6 months after discharge. Furthermore, all study patients were invited to a follow up between 3.5 and 4.5 years after the initial hospital stay. During the follow-up visit, their development quotients were determined as follows: the youngest children (< 2.5 years of age) were tested with the complementary Brunet Lézine tests and the Child Development Inventory [34]. For older children, the Raven’s progressive matrices, the Bell test, the memory of numbers and the Rey–Osterrieth complex figure (ROCF) tests were used. For all children, an interview was conducted with one or both of the child’s parents. The psychomotor development was recorded based on the parents’ statements and the child’s health records.

### Study data

For each child, we recorded the following variables: date of diagnosis, age, sex, length of hospital stay, body temperature at admission, dermatological manifestations, neurological manifestations, need for admission to the pediatric intensive care unit (PICU), history of neurological disorders, as well as variables related to CHIKV infection, namely pain level as reported using the French EVENDOL scale or self-evaluated (by visual analog scale, VAS) depending on the child’s age, arthralgia, laboratory parameters and follow-up data (head circumference, progress of child’s schooling). All data were retrospectively retrieved from the medical records.

### Criteria for defining and grading severity

The severity of the initial clinical neurological manifestations was classified into three categories, namely severe, intermediate or non-severe according to the definitions of the International Encephalitis Consortium [35].The major criterion was the presence of consciousness disorders, while minor criteria were as follows: fever > 38°C within 72 hours before or after consciousness disturbances, generalized or partial convulsive seizures not related to pre-existing epilepsy, new onset of focal neurological signs, presence of ≥ 5 leukocytes/mm^3^ in the CSF, imaging or electroencephalogram (EEG) abnormality related to encephalitis.

Manifestations were deemed to be severe in the event of: (1) confirmed or probable encephalitis (major criterion and ≥ 3 minor criteria); (2) possible encephalitis (major criterion + 2 minor criteria); (3) other systematized neurological impairment. Severity was deemed to be intermediate in case of non-encephalitic-CHIKV-associated central nervous system disease (NECACD), defined by the presence of either (1) major criterion + 1 minor criterion, or (2) major criterion alone, or (3) 2 minor criteria except fever. In the absence of these parameters, the presentation was qualified as non-severe.

### Laboratory procedures

At admission, prospective blood CHIKV RT-PCR was performed for all patients between day 1 and day 7 after disease onset. Measurement of CHIK viral load was performed retrospectively on a frozen aliquot.

As the dengue epidemic was winding down when CHIKV emerged, patients were also tested for dengue RNA, at the start of the epidemic and subsequently only in severe cases. Nucleic acid extraction was performed on 200 µL plasma or CSF with an elution volume of 50 µl using EasyMag Nuclisens from BioMérieux (Meylan, France). For detection of viral RNA, we used the RealStar Chikungunya RT-PCR kit 1.0 (Altona Diagnostics, Hamburg, Germany) with amplification on an *Applied Biosystems7500* Real-Time PCR System (Thermo Fisher Scientific, USA) and Simplexa Dengue RT-PCR assay (Focus Diagnostics, Cypress, CA) on a 3M Focus system.

Lumbar puncture was performed on admission or at the onset of neurological symptoms. The same technical procedures for CHIKV RT-PCR and dengue RT-PCR were applied to the CSF, with additional detection of herpes simplex virus and varicella zoster virus, using RealStar alpha Herpesvirus PCR 1.0 kit from Altona Diagnostics (Hamburg, Germany) and enterovirus, using ENTEROVIRUS R-gene from Argene (France).

For extraction/amplification of RNA, negative and positive controls (strain dilutions) were included in every round. The University Hospital virology laboratory participates every year in the annual program for External Quality Assessment (EQA)/ Proficiency Testing (PT) from Quality Control for Molecular Diagnostics (QCMD) (Scotland, UK), for all the viruses considered in the present study.

For measurement of viral load, RT-PCR was performed using serially diluted RNA standards with RealStar Chikungunya RT-PCR kit. The cycle threshold (Ct) values obtained were plotted against the log dilution of the plasmid to generate the standard curve. VL in the blood and CSF are presented on a log scale. The ratio was defined as. log CSF VL (copies/mL)/ log blood VL (copies/mL).

### Statistical analysis

For all descriptive and inferential analyses, the normality of the data distribution was tested. Means and standard deviations are reported for normally distributed variables. For non-normally distributed variables data are reported as median (minimum (min)-maximum (max) or interquartile range). Categorical variables are reported as number and percentage. The following tests were used for group comparisons when appropriate: Student t test, Wilcoxon-Mann-Whitney test, Chi square test and Fisher’s exact test. A logistic regression model was used to assess the association between neurological severity (dependent variable) and viral load in CSF (independent variable), adjusted for age and time between onset of clinical symptoms and lumbar puncture. Only patients with non-hemorrhagic CSF were included. The level of statistical significance was set at p < 0.05. All statistical analyses were performed using SAS version 9.4 (SAS Institute Inc., Cary, NC, USA).

## Results

During the 2014 outbreak, 1,254 children were initially suspected of having CHIKV infection. Among these, 651 were confirmed positive via blood RT-PCR. A total of 92 of these confirmed cases required lumbar puncture, of which 86 were ultimately included in the final study cohort after exclusions (Fig 1). For CSF viral load analysis, this cohort was further refined to 53 patients with non-hemorrhagic samples and a delay of less than 24h between LP and blood draw.

The sociodemographic and clinical characteristics of the patients during hospitalization for CHIKF are summarized in Table 1.

**Table 1:** Baseline sociodemographic and clinical characteristics of the patients at admission (N = 86).

Characteristic	All patients	<3 months	3 months-1 year	1-5 years	> 5 years	p-value
	(N = 86)	(N = 33)	(N = 25)	(N = 10)	(N = 18)	
Female gender	40/86 (46.5)	14 (42.4)	15 (60.0)	3 (30.0)	8 (44.4)	0.37
Length of hospital stay, days	4.7 ± 2.1	5.8 ± 2.6	4.2 ± 1.3	4.7 ± 2.3	3.5 ± 1.0	0.001
days to hospitalization after disease onset	0.6 ± 0.9	0.5 ± 0.8	0.3 ± 0.5	0.3 ± 0.5	1.1 ± 1.3	0.015
Main Cause of hospitalization						<0.001
Fever	51/86(59.3)	31 (93.9)	18 (72.0)	1 (10.0)	1 (5.6)	
Neurological disorders	35/86 (40.7)	2 (6.1)	7 (28.0)	9 (90.0)	17 (94.4)	
Body temperature at admission, °C	39.0 ± 0.7	38.8 ± 0.6	39.2 ± 0.7	39.3 ± 0.6	38.9 ± 0.8	
Other main symptoms						
Rash/Exanthema	45/86 (52.3)	27 (81.8)	11 (44.0)	5 (50.0)	2 (11.1)	<0.001
Arthralgia	14/68 (21.5)	4 (18.2)	1 (4.4)	2 (25.0)	7 (46.7)	0.01
Vomiting	11/86 (12.8)	3 (9.1)	3 (12.0)	2 (20.0)	3 (16.7)	0.75
Diarrhea	9/86 (10.4)	5 (15.2)	2 (8.0)	1 (10.0)	1 (5.6)	0.92
Pediatric intensive care hospitalization	23/86 (26.7)	17 (51.5)	2 (8.0)	3 (30.0)	1 (5.6)	<0.001
Volume loading	15/86 (17.4)	11 (33.3)	2 (8.0)	1 (10.0)	1 (5.6)	0.03
Ventilation	10/86 (11.6)	9 (27.3)	1 (4.0)	0 (0.0)	0 (0.0)	
History of neurological disorders						<0.001
None	68/86 (79.1)	31 (93.9)	23 (92.0)	5 (50.0)	9 (50.0)	
CSF (febrile seizure)	9/86 (10.5)	0 (0.0)	0 (0.0)	4 (40.0)	5 (27.8)	
Epilepsy	1/86 (1.2)	0 (0.0)	0 (0.0)	0 (0.0)	1 (5.6)	
Other	8/86 (9.3)	2 (6.1)	2 (8.0)	1 (10.0)	3 (16.7)	
History of prematurity						0.45
<32 WA	2/86 (2.3)	0 (0.0)	2 (8.0)	0 (0.0)	0 (0.0)	
32-36 WA	8/86 (9.3)	5 (15.2)	2 (8.0)	0 (0.0)	1 (5.6)	
Not premature	76/86 (88.4)	28 (84.9)	21 (84.0)	10 (100.0)	17 (94.4)	
Neurological signs and symptoms at admission						
Headache	11/28 (39.3)	***	***	2 (20.0)	9 (50.0)	***
Hypotonia	6/86 (7.0)	2 (6.1)	3 (12.0)	0 (0.0)	1 (5.6)	0.77
Meningeal syndrome	3/86 (3.5)	1 (3.0)	1 (4.0)	0 (0.0)	1 (5.6)	0.73
Simple febrile seizure	15/86 (17.4)	1 (3.0)	3 (12.0)	0 (0.0)	11 (61.1)	<0.001*
Complexe febrile seizure	15/86 (17.4)	1 (3.0)	3 (12.0)	8 (80.0)	3 (16.7)	<0.001*
Prolonged	6 (7.0)	0 (0.0)	1 (3.0)	4 (40.0)	1 (5.6)	0.001
Recurrent	8 (9.3)	1 (3.0)	2 (8.0)	3 (30.0)	2 (1.1)	0.07
Unilateral	1 (1.2)	0 (0.0)	0 (0.0)	1 (10.0)	0 (0.0)	
Generalized convulsive status epilepticus	1/86 (1.2)	0 (0.0)	0 (0.0)	1 (10.0)	0 (0.0)	
Consciousness disorders	9/86 (10.5)	1 (3.0)	2 (8.0)	2 (20.0)	4 (22.2)	0.09*
Behavioral disorders	1/86 (1.2)	0 (0.0)	0 (0.0)	0 (0.0)	1 (5.6)	0.33
Balance disorders	2/86 (2.3)	0 (0.0)	0 (0.0)	2 (20.0)	0 (0.0)	0.01*
Neurofocal signs	1/86 (1.2)	0 (0.0)	0 (0.0)	1 (10.0)	0 (0.0)	0.12
Other neurological signs	2/86 (2.3)	1 (3.0)	0 (0.0)	1 (10.0)	0 (0.0	
Abnormal medical Imaging during hospitalization						
EEG	21/55 (38.2)	7 (31.8)	3 (30.0)	4 (50.0)	7 (46.7)	0.66
TFE	0/2 (0.0)	0 (0.0)	0 (0.0)			
CT Scan	0/3 (0.0)	0 (0.0)	0 (0.0)	0 (0.0	0 (0.0	
MRI	0/7 (0.0)	0 (0.0)	0 (0.0)	0 (0.0)	0 (0.0)	
Diagnosis at discharge						<0.001*
Proven/Probable or possible encephalitis	7/86 (8.1)	1 (3.0)	1 (4.0)	2 (20.0)	3 (16.7)	0.11*
NECACD	13/86 (15.1)	2 (6.1)	1 (4.0)	3 (30.0)	7 (38.9)	0.002*
HS	14/86 (16.3)	0 (0.0)	5 (20.0)	3 (30.0	6 (33.3	0.001*
CHIKV infection without neurological severity	52/86 (60.5)	30 (90.9)	18 (72.0)	2 (20.0)	2 (11.1)	<0.001*
Severity						0.001*
Non severe	67/86 (77.9)	30 (90.9)	23 (92.0)	5 (50.0)	9 (50.0)	<0.001*
Intermediate	11/86 (12.8)	2 (6.1)	1 (4.0)	2 (20.0)	6 (33.3)	0.015*
Severe	8/86 (9.3)	1 (3.0)	1 (4.0)	3 (30.0)	3 (16.7)	0.031*

Abbreviations: WA: weeks of amenorrhea; mth: months; yr: year, EEG, electroencephalogram, TFE transfontanellar echography, CT, computed tomography, MRI, magnetic resonance imaging, NECACD: Nonencephalitic Chikungunya virus–associated CNS disease, HS: Hyperthermic seizure;

Results are presented as mean ± standard deviation, and as absolute value (percentage) for categorical variables; * Statistical significance (p < 0.05), but clinical significance of age difference is not relevant due to a selection bias related to reason to achieve lumbar puncture; ***this only concern children who are old enough to express themselves.

The sex ratio (F/M) was 0.87. All patients were admitted to hospital at the acute phase of infection, a median of 0.6 ± 0.9 days after onset of illness. All children had fever at admission. Poor fever tolerance (“septic” aspect) was the main cause of hospitalization for 51 (59.3%) patients, most of whom were less than one year old. The cause of hospitalisation for the remaining 35 patients (40.7%), mainly older children, was neurological manifestations. Of the 55 EEG performed, 21 showed a pathological pattern, including 7 slowing waves and paroxysmal peaks.

Biological findings at admission are presented in Table 2.

**Table 2:** Blood and Cerebrospinal fluid biological characteristics at admission.

Characteristics	All patients (N = 86)	< 3 months (N = 33)	3 months-1 year (N = 25)	1-5 years (N = 10)	> 5 years (N = 18)	p-value
**Blood**						
White cells, G/L^N = 73^	8.0 ± 3.5	6.9 ± 3.3	9.1 ± 3.8	10.3 ± 2.9	7.2 ± 2.8	*
Lymphocytes, G/L	1.1 ± 0.9	1.0 ± 0.7	1.5 ± 1.0	1.5 ± 1.2	0.6 ± 0.3	*
Platelets, G/L	280.1 ± 115.9	306.7 ± 126.5	317.1 ± 123.4	240.6 ± 62.4	201.7 ± 51.3	<0.0001
Leucopenia ^N = 73^	15 (20.6)	9 (32.1)	4 (18.2)	0 (0.0)	2 (13.3)	0.22
Lymphopenia	76 (88.4)	31 (93.9)	20 (80.0)	7 (70.0)	18 (100.0)	0.03
Prothrombin level, ^N = 65^	76.5 ± 16.1	80.8 ± 16.1	74.4 ± 18.6	79.8 ± 11.3	69.7 ± 12.8	0.29
C-reactive protein, mg/L	21.0 ± 24.9	13.3 ± 15.8	30.7 ± 30.7	14.7 ± 16.3	25.2 ± 29.1	0.0004
ASAT IU/L^N = 71^	47.2 ± 51.7	61.4 ± 81.3	42.3 ± 14.7	41.3 ± 10.7	31.5 ± 7.1	0.02
ALAT IU/L^N = 71^	22.4 ± 25.7	29.1 ± 39.5	21.8 ± 9.6	17.4 ± 12.3	13.9 ± 5.5	0.003
CHIKV VL in blood^#^	9.8 ± 1.2	10.6 ± 0.8	10.0 ± 0.5	9.4 ± 0.7	8.4 ± 1.6	<0.0001
Days to blood VL assessment	0.8 ± 1.2	0.9 ± 1.2	0.4 ± 0.5	0.4 ± 0.5	1.6 ± 2.0	0.045
**Cerebrospinal fluid (CSF)**						
Positive RT-PCR	84 (97.7)	32 (97.0)	25 (100.0)	10 (100.0)	17 (94.4)	0.77
Pleocytosis ≥5^**^	5	1	0	1	3	
Glycorachy, mmol/L^**^	4.0 ± 0.7	3.6 ± 0.6	4.2 ± 0.8	3.9 ± 0.7	4.1 ± 0.6	0.08
Proteinorachy, g/L^**^	0.23 ± 0.15	0.37 ± 0.19	0.17 ± 0.08	0.15 ± 0.04	0.19 ± 0.07	*
CHIKV VL in CSF ^**^	4.90 ± 1.69	6.18 ± 1.98	5.06. ± 0.9	4.71 ± 0.87	3.17 ± 1.13	<0.0001
Ratio**	0.50 ± 0.15	0.60 ± 0.18	0.51 ± 0.08	0.49 ± 0.08	0.38 ± 0.14	<0.001

VL, viral load; copies/mL are on a log scale

Blood test results were interpreted according to the usual values for the age group

Results are presented as mean ± standard deviation or median and min-max ranges [min-max] for quantitative variables, and as absolute value (percentage) for categorical variables

* P values not calculated: normal values depend on age groups

**Thirty-three CSF excluded from the quantitative analysis: thirty were hemorrhagic and 2 LP had a delay >1 day and 1 was drawn 2 days before blood

The most common finding was lymphopenia (88.4%). In addition to CHIKV, CSF was tested for other viruses in order to rule out possible co-infection. Thirty CSF were hemorrhagic (> 100 red cells/mL) and were excluded from the quantitative analysis. Pleiocytosis (white cells ≥ 5/mL) was observed in 5 cases but never exceeded 10 white cells/mL. All CSF samples were CHIKV positive by RT-PCR, except in two children.

According to international recommendations from the International Encephalitis Consortium, 7 out of 86 (8.1%) patients were discharged with a proven/probable or possible diagnosis of encephalitis, 13 (15.1%) with NECACD, 14 (16.3%) with hyperthermic seizures (HS) and 52 (60.5%) with chikungunya infection without severe neurological manifestations (Table 1). Eight patients were classified as having severe illness, namely the 7 cases of encephalitis and one case of NECACD with cerebellar syndrome. All other NECACD cases were classified as having intermediate severity. Patients with hyperthermic seizures were deemed non-severe.

No relation was found between the severity of neurological manifestations and the VL in the blood or CSF (Table 3). By multivariable analysis, CSF viral load was not significantly associated with the severity of neurological manifestations (OR = 1.03 [95% CI 0.62, 1.85]) after adjustment for age and time between onset of clinical signs and lumbar puncture.

**Table 3:** Chikungunya viral load and CSF/blood ratio according to neurological severity.

	Non severe (N = 38)	Intermediate (N = 8)	Severe (N = 7)	P value
Blood VL (N = 86)	9.74 ± 1.26	9.56 ± 0.88	9.73 ± 1.90	0.15
CSF VL (N = 53) *	4.95 ± 1.80	4.79 ± 1.67	4.73 ± 1.13	0.93
Ratio (N = 53)**	0.50 ± 0.16	0.49 ± 0.13	0.50 ± 0.11	0.95

Results are presented as mean ± standard deviation

VL, viral load; copies/mL are on a log scale, *patients with non-hemorrhagic CSF; **ratio = log CSF VL/ log blood VL

**Fig 2:**
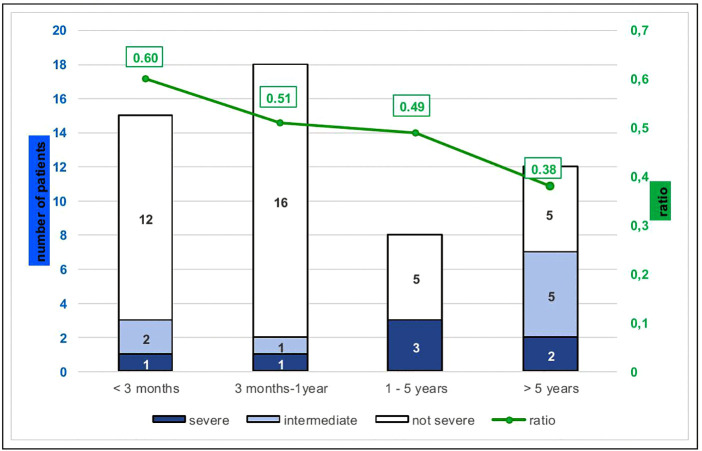
Severity of chikungunya infection according to age and ratio values. ratio values = log (CSF VL)/log (blood VL). VL: Chikungunya viral load.

To better visualize the results, a ratio (log (CSF VL)/log (blood VL)) was calculated for the non-hemorrhagic CSF (Fig 2). The mean ratio was 0.50 [0.0-0.73].

No death occurred in the study population during hospitalization. It should be noted that none of the children had sickle cell disease.

One to six months (2.0 ± 1.6) after discharge, twenty children (aged < 3 months or with initial severe manifestations such as encephalitis) had follow-up. The results of the medical consultation and the normal imaging findings showed a good apparent recovery.

A late neurological follow up was also scheduled for all patients. Overall, 45 patients (60% aged < 1 year) were seen between 3.5 and 4.5 years (4.1 ± 2.23) after the initial infection phase. No response was obtained from the parents of the 41 remaining patients, so they were considered lost to follow-up.

Three of the 18 children aged >1 year at the time of diagnosis, and 5/27 patients aged <1 year at the time of diagnosis presented with neurological impairment, even though the latter group had not been explored because of the neurological manifestations, but rather, due to poor tolerance of fever (Table 4).

**Table 4:** Summary of neurological characteristics observed at long term follow-up.

	Number of children (N = 27*)	Number of children (N = 18**)
abnormal psychomotor development according to parents	3	3
linguistic delay	2	2
Global retardation	1	1
School performance		
difficulties	NA	6
Need for specific support	1	1
head circumference		
> +2 SD	0	3
+2 SD > z score > +1 SD	0	6
+1 SD > z score > -1 SD	21	9
-1 SD > z score > -2 SD	5	0
<-2 SD	1	0
Neurological impairment	2	5
Dysmorphic disorder, global retardation	0	1
medial territory paresthesia	0	2
Visual defects	0	1
Contact difficulties	0	1
Pyramidal syndrome and nystagmus	1	0
Echolalia and stereotypies	1	0

* Children who were infected with chikungunya when they were less than 1 year of age (mean age at long term consultation 4.4 ± 0.3 years)

** children who were infected with chikungunya when they were 1 year of age or over (mean age at long term consultation 9.7 ± 2.8 years)

In children with long-term neurological disorders, the mean log VL was 4.6 ± 1.3 in the CSF, and 9.6 ± 1.3 in the blood. For the remaining patients without neurological impairment, it was 5.7 ± 2.3 and 9.8 ± 1.3 respectively. In terms of viral load, there was no statistical difference between the children with long-term neurological signs and those without (p = 0.24 for CSF and 0.70 for blood).

In all, 3 of the 45 children showed developmental delays requiring more in-depth neuropsychological assessment and rehabilitation.

## Discussion

### Clinical features at admission

This single-center, observational, longitudinal, retrospective study reports the neurological manifestations observed in children hospitalized during the 2014–2015 CHIKV epidemic in Martinique, and analyzes the relation between CHIKF disease severity and the viral load in the blood and CSF.

All children had fever and were admitted to hospital at the acute phase of infection. Neurological manifestations were the main cause of hospitalization in 40.7% of the children, mainly those older than one year. The onset of neurological signs was concomitant with the onset of fever in 84.9% of the children in our study, which is similar to the 73% reported in the study from Reunion Island by Robin *et al.* [8,13]*.* Seizures are common in children, varying from 12% [14] to 56.3% [19]. In the present study, seizures occurred in all age ranges, and was the main reason for lumbar puncture in children aged over 1 year (22 out of 28).

The leading causes of PICU admission in our cohort were severe neurological manifestations (26.7%), very young age and/or poorly tolerated fever or pain, in line with the 24% and 37.9% respectively described elsewhere [8,36]. A septic aspect was the leading reason for lumbar puncture in children younger than 3 months.

### Biological parameters, EEG and neuroimaging

The lymphopenia, normal or slightly elevated proteinorachia and low or absent pleocytosis observed in our study are common in CHIKV-infected infants [8,11,13,37]. Slowing waves and paroxysmal peaks, observed on the 21 abnormal EEG of our study patients, and the unremarkable results of the neuroimaging were similar to observations in other studies [8,16].

### Viral loads

During the first days of illness, the VL of CHIKV in the blood is known to be very high [26,38,39]. It depends on age and is higher in newborns and older adults [16,40]. Our results (mean VL in the blood > 9 log) are not unexpected, since all the children were hospitalized early after the onset of clinical signs. Moreover, blood VL varied significantly across age groups, with infants younger than 3 months presenting with the highest VL (p < 0.0001).

The possible association between blood VL and clinical manifestations and/or disease severity remains contradictory [41,42]. We found no clear relationship between blood VL and the clinical presentation of children, similar to the findings from an Indian study [26]. This suggests that the blood viral load may not contribute significantly to the severity of CHIKV infection.

In addition to blood screening, the presence of CHIKV RNA in the CSF has also been studied as a means to explore the virological basis of neurological manifestations” [8,13], In our study, all CSF samples were positive except for two cases, probably due to delayed hospitalization (>3 days after symptom onset) in one case; the cause remains unexplained for the other.

CSF VL in CHIKF patients has been reported to be related to plasma VL and is generally lower [16,28]. The results of this study are in agreement with those found in a cohort of encephalitis and NECACD in Reunion Island [15]. As with plasma VL, CSF VL was significantly higher in patients aged less than 3 months (p < 0.001) in our study cohort. These findings further report that blood VL was on average 4.5x10^5^ higher than CSF VL. The ratio (CSF VL/Blood VL) varied across age groups. Since the time between lumbar puncture and blood sampling was ≤ 24h, and hemorrhagic CSF samples were excluded from calculation, these findings suggest that there might be passive diffusion of viral RNA from the blood to the CSF, rather than intrathecal replication. This hypothesis should have been further explored by assessing cytokines and intrathecal antibody response, but this could not be done given the small quantity of CSF available and the retrospective study design.

### Severity of the neurological manifestations and outcome

Seven of the 86 (8.1%) children included in this study developed encephalitis; when compared with the 651 positive cases diagnosed in hospital, this rate fell to 0.1%.

These results seem less worrying than those reported from Reunion Island, [8,16]. Among children hospitalized for CHIKF in Honduras in 2015, it was reported that 6% had meningoencephalitis [18]. CHIKV strains are different, with an Asian lineage in Martinique and the IOL strain in Reunion Island, but likely, the same strain in Martinique and Honduras. However, a possible difference in neurovirulence between ECSA and Asian strains cannot be ruled out. This question remains to be studied by comparing the clinical and virological data of patients with severe neurological signs associated with CHIKV. Patient recruitment was also different, since newborns were excluded from our study, and all the children had CHIKV infection proven by positive RT-PCR in the blood and concomitant screening in the CSF at the acute phase of infection.

Severity does not seem to be related to neurological history, since no encephalitis occurred in the 18 children who had previous neurological manifestations.

VL and ratios were not significantly different in the 3 groups (severe, intermediate and non-severe), as in the study of Gerardin et al. [16]*,* where they studied a cohort including infants and adults. In our study, children aged over one year accounted for up to 75% of severe cases, whereas they represented only 32.6% of the cohort. The differences in severity according to age are biased in our study since the reason for undergoing LP, and therefore for inclusion in the cohort, was different for infants and for children over one year of age. However, at any age, the severity of neurological manifestations did not differ according to the VL or the ratio.

Our study showed an almost constant spread of CHIKV in the CSF, raising the question of possible cognitive sequelae in children. Severe cognitive disorders were observed in newborns infected during the epidemic on the Island of Réunion [22].

Our preliminary study of the children’s psychomotor development is worrying, although the low number of patients, and the absence of control group precludes drawing any firm conclusions.

None of our patients died, whereas other studies reported mortality of up to 6.6% [20,22]*.* During the same epidemic, the crude mortality rate was 26% among 65 adults (10 of whom had acute neurologic manifestations) admitted to intensive care units in Martinique and Guadeloupe (a neighboring French island) [9]. These data suggest that the fatality rate of CHIKV infection seems to be less related to the viral genotype, and more to other factors, including older age and comorbidities.

The linearity of CSF VL and ratios according to age is all the more remarkable since our study includes pediatric populations from different age groups. This observation argues in favor of the hypothesis of passive diffusion of CHIKV in the CSF, and no role of intrathecal replication in the pathophysiology of neurological impairment. The latter therefore remains to be explored. The spread of the virus in the CSF has been demonstrated in a mouse model. After peripheral inoculation followed by viremia, the CHIKV can spread and invade the CNS via the choroid plexus, then spread in the CSF [32].

The great variability in the incidence of this disease during the various epidemics around the world raises two hypotheses: the role of viral variants and that of the immune specificities (genetics or cross-protection) of each population. The early onset of neurological manifestations in our study suggests, as with dengue encephalopathy, that there is a vascular mechanism secondary to primary cytokine response. The only three autopsies we found in the literature are in favor of this hypothesis [30,31].

Recently, in a study screening many cytokines, Santos Almeida and coworkers [43]. proposed the IL-22/IL-17A ratio as the best marker of neurological damage in adults infected by Arbovirus. These authors suggested that this cytokine profile promotes the breakdown of the blood-brain-barrier. A study of cytokines in the CSF could provide a better understanding of the mechanisms and treatment of neurological damage. However, since our study has shown that LP does not yield any benefit in the immediate management of children, in the future, LP will only be ethically justified for differential diagnosis and severe forms.

## Strengths and limitations

Our study has some limitations that deserve to be mentioned, including the single-center, retrospective design. Further, we included only children with positive RT-PCR on blood samples drawn at admission, and we may therefore have missed some patients with late neurological manifestations occurring more than 5 days after the onset of disease, in whom RT-PCR could be negative.

However, this limitation is also an advantage because, unlike other studies where infection was documented in blood and/or CSF by RT-PCR or IgM detection, we can assert that all patients are in the acute phase of their infection. All children who require hospitalization in Martinique for suspected infectious disease are referred to our hospital. We can therefore assume that we included the vast majority of children (except newborns) with acute CHIKV infection and neurological manifestations. Lastly, at the start of the epidemic, all infants were systematically investigated, and were hospitalized more often than later in the epidemic. In total, 52% of children attended the long-term follow-up. The remainder were considered lost to follow-up. Furthermore, the absence of a control group prevents us from drawing definitive conclusions.

## Conclusion

Neurological manifestations, including encephalitis, may complicate CHIKV infection and are a major challenge in children. Our results reveal that the virus almost always invades the CNS at a high level during the acute phase of infection. No significant association was found between the viral load in the CSF or blood, and neurological severity, either at the acute phase of the disease or in the long-term cognitive development of infected children. In contrast, viral load was inversely associated with age.

Pending worldwide implementation of the recently approved vaccine, which offers a new perspective but is not yet available for children, further research is needed to understand the pathophysiology of the neurological manifestations of chikungunya and the impact of infection on the long-term neurological development of children.
